# Left Ventricular Deformation and Vortex Analysis in Heart Failure: From Ultrasound Technique to Current Clinical Application

**DOI:** 10.3390/diagnostics11050892

**Published:** 2021-05-17

**Authors:** Simona Sperlongano, Antonello D’Andrea, Donato Mele, Vincenzo Russo, Valeria Pergola, Andreina Carbone, Federica Ilardi, Marco Di Maio, Roberta Bottino, Francesco Giallauria, Eduardo Bossone, Paolo Golino

**Affiliations:** 1 Division of Cardiology, Department of Translational Medical Sciences, University of Campania Luigi Vanvitelli, 80131 Naples, Italy; sperlongano.simona@gmail.com (S.S.); vincenzo.russo@unicampania.it (V.R.); andr.carbone@gmail.com (A.C.); ro.bottino@hotmail.com (R.B.); paolo.golino@unicampania.it (P.G.); 2 Department of Cardiology and Intensive Coronary Care, Umberto I Hospital, Nocera Inferiore, 84014 Salerno, Italy; 3 Department of Cardiac Thoracic Vascular Sciences and Public Health, University of Padua Medical School, 35100 Padova, Italy; donato.mele@unipd.it (D.M.); valeria.pergola@aopd.veneto.it (V.P.); 4 Department of Advanced Biomedical Sciences, Federico II University of Naples, 80138 Naples, Italy; fedeilardi@gmail.com; 5 Division of Cardiology, Maria SS. Addolorata Hospital, Eboli, 84025 Salerno, Italy; marcodimaio88@gmail.com; 6 Department of Translational Medical Sciences, Federico II University of Naples, 80138 Naples, Italy; giallauriafrancesco@gmail.com; 7 Division of Cardiology, “Antonio Cardarelli” Hospital, 80131 Naples, Italy; ebossone@hotmail.com

**Keywords:** heart failure (HF), heart failure with preserved ejection fraction (HFpEF), cardiac resynchronization therapy (CRT), speckle tracking echocardiography (STE), left ventricular strain, color Doppler flow mapping (CDFM), left ventricular vortex

## Abstract

Heart failure (HF) is a leading cause of cardiovascular morbidity and mortality. However, its symptoms and signs are not specific or can be absent. In this context, transthoracic echocardiography plays a key role in diagnosing the various forms of HF, guiding therapeutic decision making and monitoring response to therapy. Over the last few decades, new ultrasound modalities have been introduced in the field of echocardiography, aiming at better understanding the morpho-functional abnormalities occurring in cardiovascular diseases. However, they are still struggling to enter daily and routine use. In our review article, we turn the spotlight on some of the newest ultrasound technologies; in particular, analysis of myocardial deformation by speckle tracking echocardiography, and intracardiac flow dynamics by color Doppler flow mapping, highlighting their promising applications to HF diagnosis and management. We also focus on the importance of these imaging modalities in the selection of responses to cardiac resynchronization therapy.

## 1. Introduction

Heart failure (HF) is a clinical syndrome characterized by dyspnea, fatigue, and limitation of physical activity, due to reduced cardiac output and/or elevated ventricular filling pressures at rest or during stress. It has a prevalence of about 1–2% in the adult population of developed countries, reaching ≥10% among people >70 years of age [1]. Once developed, HF results in significant cardiovascular morbidity and mortality. More than one million people are annually hospitalized for HF, with a 5-year mortality rate of approximately 50% [2].

HF is suspected by clinical history, symptoms, physical examination, electrocardiogram findings, and natriuretic peptides’ values. However, since symptoms and signs are non-specific for HF or can be absent, echocardiography plays an essential role in supporting HF diagnosis. Transthoracic echocardiography (TTE), because of its easy application, non-invasive nature and safety, is the method of choice to assess myocardial structure and function and establish the diagnosis of HF with reduced, mid-range and preserved ejection fraction (HFrEF, HFmrEF, and HFpEF, respectively) (Class I of recommendation, according to European guidelines on HF1). In addition to diagnosis, TTE is a key tool in guiding therapeutic choices and monitoring response to treatment [1].

Over the last few decades, several ultrasound modalities have been introduced in the field of echocardiography, aiming at better understanding the morpho-functional abnormalities occurring in cardiovascular diseases. These new modalities are still struggling to enter the clinical practice. This review article deals with some of these new cardiac ultrasound technologies, in particular analysis of myocardial deformation and intracardiac flow dynamics, highlighting their promising applications to HF diagnosis and management.

## 2. Left Ventricular Speckle Tracking Echocardiography

### 2.1. Left Ventricular Mechanics

During systole, left ventricular (LV) myocardium undergoes a three-dimensional (3D) deformation, characterized by radial thickening and longitudinal and circumferential shortening. An additional movement of rotation of the LV around its long-axis is present: viewed from apex, the apical rotation is anticlockwise while the base rotation is clockwise. The resulting motion is called twist and is essential for LV ejection. During diastole, the untwist motion occurs, which generates a suction force driving the early, rapid LV diastolic filling [3].

This complex 3D deformation during the cardiac cycle is allowed by the double-helical orientation of the LV myocardial cells. In particular, the endocardial helix, which is more parallel to the LV long-axis, is involved mainly in longitudinal deformation, whereas the epicardial helix is implicated primarily in circumferential shortening. Radial thickening and LV rotational motion are permitted by the contribution of both subendocardial and subepicardial fibers.

### 2.2. Left Ventricular Speckle Tracking Echocardiography: The Technique

Two-dimensional (2D) speckle tracking echocardiography (STE) is a gray-scale based technique which allows the assessment of myocardial deformation independently from insonation angle. All the above-mentioned components of the LV contractile function can be evaluated by STE [4]. Therefore, STE overcomes the two main limitations of tissue Doppler deformation imaging, the heavy dependence on insonation angle, and the possibility of analyzing only longitudinal deformation.

Myocardial deformation can be quantified as strain or strain rate. Strain is the difference between the length of a myocardial segment after contraction and its resting length, expressed as a percentage; strain rate is the rate at which this deformation takes place, expressed as 1/s. Normally, strain and strain rate values are negative in systole when myocardium shortens, and positive in diastole when myocardium lengthens.

A number of bright speckles generated by the interaction of the ultrasound beam with the myocardium and its subsequent scatter, are identified by the STE software and followed frame-by-frame throughout the cardiac cycle. Then, by using an algorithm, the software calculates the magnitude of myocardial deformation in each direction and generates strain and strain rate curves. Today, strain analysis can be performed either online at the patient bedside, or offline on a workstation, and even with portable devices.

**Figure 1:**
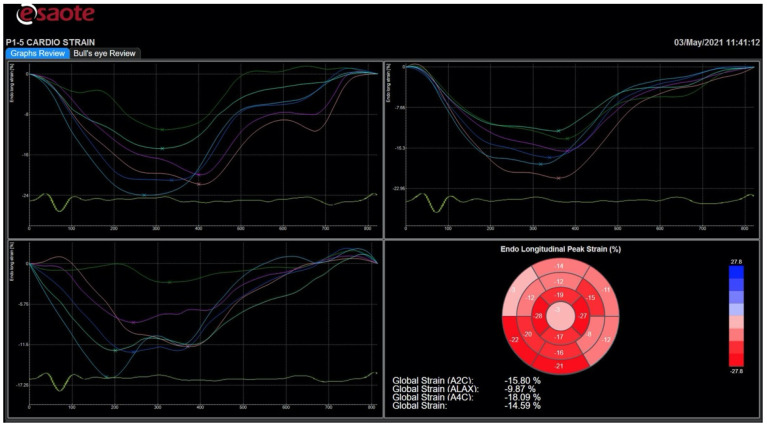
Strain curves and bull’s eye in a patient with heart failure and reduced ejection fraction. Scheme 3. 4-, and 2-chamber views are not homogeneous, indicating that left ventricular segments do not contract all at the same time, and also the degree of systolic shortening is greater in some segments and impaired in others. At the bottom right, the bull’s eye displays a reduced value of global longitudinal strain. All images were obtained using an Esaote Mylab echo-scanner.

To measure the LV longitudinal strain, high quality images should be taken from the apical four-chamber, two-chamber and long-axis view, with the LV occupying most of the sector and without LV foreshortening. A frame rate between 30 and 70 frames/s is optimal for images’ acquisition. ECG-gating is mandatory, permitting to collect three cardiac cycles for each view. The timing of aortic valve closure is essential in the deformation study; therefore, it is recommended to start speckle tracking analysis from the apical long-axis view, where aortic valve leaflets motion is displayed. The endocardial borders are manually traced in the end-systolic frame automatically generated by the software. Then, the software brings up a region of interest (ROI), including the entire myocardial thickness, which can be manually modified in width. Subsequently, myocardial speckles are automatically tracked frame-by-frame. If tracking is not adequate, the operator can adjust the ROI. Once the operator approves the tracking, LV myocardium is divided by the software in six segments, and segmental and global longitudinal strain, myocardial velocities, and strain curves are provided (Figure 1). The whole process needs to be repeated for the apical four- and two-chamber views in order to obtain strain values for all myocardial segments, and their average, the LV global longitudinal strain (GLS). The most recent echo scanners have fully automated the process, which is now based on artificial intelligence, and also allow automated recognition of the three apical views needed for the analysis. Strain data can be displayed through a bull’s eye plot, which intuitively shows segmental and global longitudinal strain values (Figure 1) [5]. Information about circumferential and radial deformation, and LV twist can be obtained from LV short-axis views.

Normal values of LV strain have been derived from a meta-analysis [6], even if inter-vendor variability, based on the use of different approaches to speckle tracking calculation of strain by different vendors, was observed for radial strain in particular. A recent European, large, multicenter, prospective study confirmed inter-vendor (GE and Philips) variability for radial and circumferential strain despite the use of a vendor-independent software, whereas no inter-vendor difference was observed for longitudinal strain [7].

### 2.3. Clinical Application of Left Ventricular Speckle Tracking Echocardiography to Heart Failure with Preserved Ejection Fraction

According to the task force for the diagnosis and treatment of acute and chronic HF of the European Society of Cardiology (ESC), STE-derived indices should be considered (class IIa recommendation) in a TTE protocol for subjects at risk of developing HF in order to identify myocardial dysfunction at the preclinical stage, when LVEF is still normal [1].

LV longitudinal systolic dysfunction identified by STE imaging is common among patients affected by HFpEF [8,9,10]. This is because HFpEF comorbid conditions, such as type 2 diabetes, systemic arterial hypertension, history of coronary artery disease, obesity, and severe LV hypertrophy, often cause interstitial fibrosis, which primarily involves LV subendocardial fibers [11]. Therefore, HFpEF is now considered a disorder characterized, not only by isolated LV diastolic dysfunction, but also by LV systolic longitudinal abnormalities.

The isolated impairment of the subendocardial layer is compensated by the augmentation of function of the other layers, so that LVEF and overall LV performance remain preserved [2,11]. Thus, longitudinal systolic dysfunction in patients with HFpEF is counteracted by a normal rotational, circumferential and radial LV contraction. A GLS value < 16% currently falls within the minor criteria of HFpEF workup and scoring system, according to a consensus document drafted by the HF Association of the ESC [12].

Beyond its diagnostic role, GLS also seems to play a prognostic role in HFpEF. Impaired GLS predicts HF hospitalization, cardiovascular death, or cardiac arrest [13,14]. Moreover, lower GLS values are associated with higher NT-proBNP levels, which is a proven prognostic factor in HFpEF [8]. Further and larger multicenter studies are needed to confirm the role of STE in identifying patients with HFpEF who are at particularly high risk for cardiovascular morbidity and mortality.

Finally, abnormal GLS during exercise has been found to be an independent predictor of the occurrence of all-cause death and HF hospitalization in patients with HFpEF [15]. Probably, LV longitudinal systolic dysfunction can favor the insufficient rise of stroke volume and cardiac output during exercise, mechanism which may contribute to reduced functional capacity during effort in patients with HFpEF [11].

### 2.4. Clinical Application of Left Ventricular Speckle Tracking Echocardiography to Heart Failure with Reduced Ejection Fraction

In the HFrEF setting the diagnostic value of STE-derived parameters is apparently less important as in HFpEF, since LV contractile function is clearly impaired. However, speckle tracking imaging plays a significant role in risk stratification and decision making of patients with HFrEF.

GLS is an independent predictor of all-cause mortality in HFrEF patients, superior to standard echocardiographic parameters [16]. Moreover, it has been shown to be an accurate predictor of ventricular arrhythmias in patients with reduced LVEF [17]. In patients with acute HF, GLS has greater prognostic value than LVEF during a 5-year follow-up period [18].

Finally, strain represents a practical tool to assess early variations of LV systolic function during follow-up of patients who start new therapies. A recent study proved that GLS was able to capture the early benefit of sacubitril/valsartan on LV remodeling in patients with HFrEF after three months of treatment, when LVEF was not significantly changed [19]. In a large study conducted on patients with LV dysfunction or HF after myocardial infarction, circumferential strain rate, but not longitudinal strain rate, was strongly predictive of LV remodeling at 20 months of follow-up [20].

In Table 1 the patterns of deformation imaging and their role in different HF phenotypes are reported.

## 3. Left Ventricular Color Doppler Flow Mapping

### 3.1. Left Ventricular Vortices

Normally, during diastole, when blood flow enters the LV from the left atrium, two vortical structures appear in an echocardiographic apical long-axis view, which rotate around a virtual axis, storing kinetic energy: the main structure is anterior and rotates clockwise, the other is smaller, posterior and rotates counterclockwise. This vortical flow configuration is mainly related to the natural asymmetric geometry of the mitral valve apparatus (the anterior leaflet is longer than the posterior, and the mitral valve orifice is eccentric as compared with the LV longitudinal axis). Studies based on magnetic resonance imaging evidenced that a vortex ring with toroidal shape is present in the LV around the tip of the mitral valve leaflets. Therefore, the two vortical structures described above are actually the result of sectioning the vortex ring with a bidimensional echocardiographic plane.

During the cardiac cycle, the vortex flow changes. In particular, during the isovolumic contraction phase, blood is redirected toward the LV outflow tract, with formation of a large anterior vortex across the LV inflow-outflow region; then, when aortic valve opens, blood is ejected.

Analysis of intracardiac flow dynamics is a way to approach the study of the LV contractile function [21].

### 3.2. Color Doppler Flow Mapping: The Technique

Phase-contrast magnetic resonance imaging is considered the gold standard for measuring intracardiac flow dynamics in heart cavities. Echocardiography can also assess intracardiac flow using various modalities. The echocardiographic particle image velocity (Echo-PIV) technique requires the administration of an echocardiographic contrast medium. A different approach is based on color Doppler flow mapping (CDFM) and does not require contrast administration. Currently, there are two commercially available techniques that allows qualitative and quantitative evaluation of vortex flow using standard echo scanners and adult transducers: the HyperDoppler technique (by Esaote) and the Vector Flow Mapping technique (by Hitachi Medical System).

**Figure 2:**
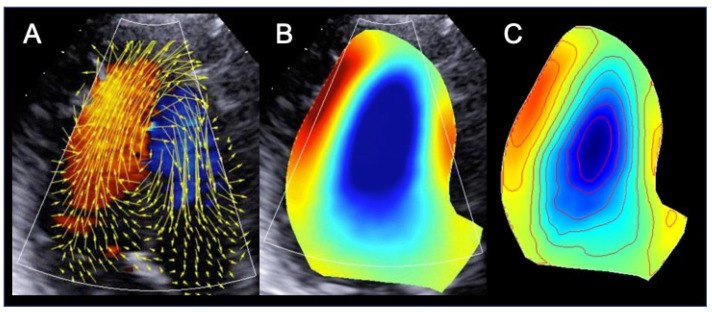
Intracardiac flow dynamics in a patient with dilated cardiomyopathy. Different maps were used to represent flow properties. The vector flow map (**A**) shows that the flow circulates along the posterolateral wall and is rotating anteriorly at the level of the left ventricular apex. In the circulation map (**B**) this translates into the formation of a single large vortex, that rotates clockwise (blue color) at the mid-apical portion of the left ventricle. The steady-streaming flow map of one heartbeat (**C**) shows the streamlines and the color map of the vorticity field. All images were obtained using the HyperDoppler software of an Esaote Mylab X8 echo-scanner without contrast injection.

**Figure 3:**
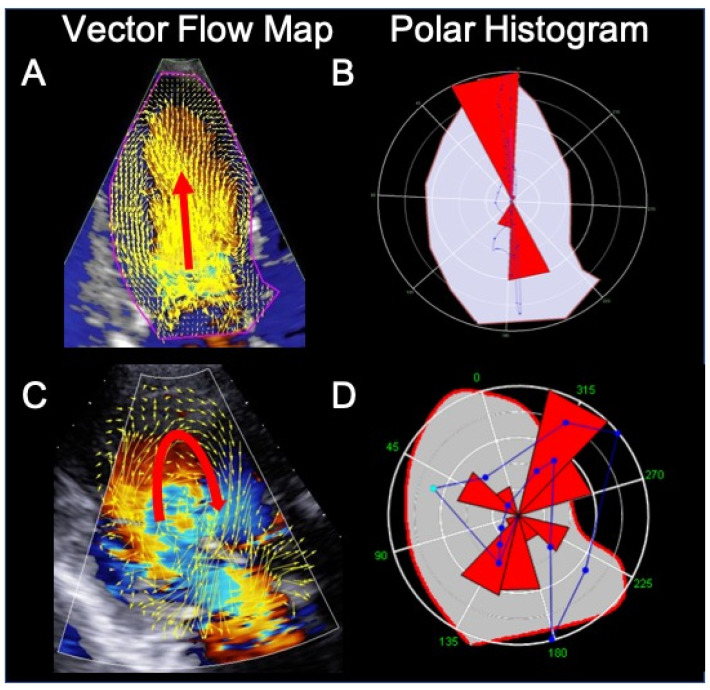
Intracardiac flow analysis of a normal subject (**A**) and (**B**) and a patient with dilated cardiomyopathy (**C**) and (**D**). In the normal subject, during diastole, left ventricular (LV) filling occur mainly along a longitudinal axis (panel (**A**), red arrow). Panel (**B**) shows the intensity-weighted polar histogram representing the distribution and intensity of the LV hemodynamic forces occurring during the entire heartbeat. The hemodynamic forces (in red) are aligned along the LV base–apex direction according with the normal emptying–filling process of the LV. In the cardiomyopathy patient, LV filling is abnormal, with flow circulating along the posterolateral wall and rotating anteriorly at the level of the left ventricular apex (panel (**C**), red arrow). The intensity-weighted polar histogram (panel (**D**)) shows a dispersed distribution of the intraventricular hemodynamic forces. Images were obtained using the HyperDoppler software of an Esaote Mylab X8 echo-scanner without contrast injection.

The HyperDoppler technique provides different possibilities to analyze and represent intracardiac flow data: a flow velocity vector map, where velocity vectors are displayed as arrows superimposed on the traditional color Doppler flow images; a circulation parametric map, where vortices are represented as compacted regions in blue (clockwise rotation) or in red (counterclockwise rotation); and a steady-streaming flow map of one heartbeat, which can be used for a number of quantitative measures, including vortex area, length, depth, and intensity (Figure 2). In addition, the software allows to evaluate kinetic energy dissipation or loss within the LV and intraventricular hemodynamic forces. While a normal LV shows longitudinal alignment of the intraventricular hemodynamic forces, in a dilated and dysfunctional LV, transversal hemodynamic forces also occur, with dispersion of their distribution (Figure 3).

### 3.3. Clinical Application of Color Doppler Flow Mapping to Heart Failure

The LV vortex formation arises from an optimal interaction between LV chamber geometry, mitral valve apparatus morphology, and normal electrical conduction system, which allows the harmonic, synchronous contraction of the cardiac walls [20]. The alteration of one of these elements affects the LV vortex formation.

In dilated cardiomyopathy, during diastole, a single vortex is generally located in the center of the LV cavity, which is larger, rounder, and more persistent than in normal subjects, with a greater amount of kinetic energy [22,23,24]. Kinetic energy dissipation is higher in healthy subjects than in patients with dilated cardiomyopathy or myocardial infarction and impaired LVEF and stroke volume [25,26]. Finally, larger infarctions are associated with a more severe alteration in LV intracavitary blood flow dynamics [27]. Preliminary data reported the prognostic value of vortex properties in patients with HF [28,29,30,31].

## 4. New Ultrasound Technologies for Cardiac Resynchronization Therapy

Cardiac resynchronization therapy (CRT) is currently recommended in class I by the Task Force on cardiac pacing and resynchronization therapy of the ESC in chronic HF patients with LVEF ≤35% and New York Heart Association (NYHA) functional class II–IV despite medical treatment, when a left bundle branch block (LBBB) is present with QRS duration ≥130 ms in sinus rhythm [32]. Among these patients, CRT has proven its beneficial effect on symptoms, HF hospitalizations, and mortality [33,34,35,36,37].

The indication for CRT implantation in patients with sinus rhythm and non-LBBB morphology is more equivocal: class IIa in patients with QRS duration >150 ms and class IIb in patients with QRS duration 130–150 ms. CRT is not indicated in patients with QRS duration <130 ms (class III) [32].

In the field of echocardiography, there was a significant effort in developing methods aiming at assessing mechanical intraventricular dyssynchrony, and identifying patients with the greatest potential benefit from CRT implantation [38,39].

The multicenter, observational PROSPECT study proved that indices of LV dyssynchrony obtained with conventional echocardiography and tissue Doppler imaging do not predict CRT success better than ECG among patients with NYHA III-IV HF, LVEF ≤ 35%, and QRS ≥ 130 ms, and that their clinical applicability is too low for routine practical use [40]. After the PROSPECT trial, more advanced ultrasound technologies have provided new measures of LV mechanical dyssynchrony.

Using 2D STE and radial strain analysis, it is possible to calculate the anteroseptal-posterior wall delay (ASPWD) in parasternal short-axis view at the papillary muscle level, that is, the time difference between the maximal thickening of the anteroseptal and posterior wall. This modern index of radial intraventricular dyssynchrony predicts response to CRT and long-term prognosis (HF hospitalizations and mortality) [41,42,43,44]. However, the value of ASPWD has been questioned by the negative results of the EchoCRT study, conducted on patients with systolic HF, narrow QRS complex (<130 ms), and mechanical dyssynchrony evaluated by echocardiography [45].

The mechanical dispersion index, originally defined as the time to peak longitudinal strain standard deviation [46], is another measure derived by 2D STE used to assess LV dyssynchrony (mechanical dispersion) in patients with HF undergoing CRT.

2D STE also allows to evaluate LV mechanical discoordination, defined as the coexistence of some LV segments in contraction and others in relaxation. LV mechanical discoordination is a time-independent feature which improves the prediction of CRT response rather than mechanical dyssynchrony [47,48]. By using 2D STE in patients with LBBB, it is possible to identify the typical pattern of mechanical discoordination, characterized by early, rapid contraction of the septum and simultaneous passive stretching of the lateral wall, which begins and finishes its contraction later [39,49]. This classic pattern is predictive of response to CRT [50].

The rapid, septal pre-ejection contraction, which occurs during the isovolumetric phase of systole, followed by a rapid septal elongation, is called septal flash. The delayed lateral wall contraction, which follows the septal flash and reaches its peak after aortic valve closure, causing a lateral motion of the apex, is called apical rocking. Basing on recent evidence, septal flash and apical rocking could provide added value to the current guideline criteria for CRT candidate selection [51,52].

**Figure 4:**
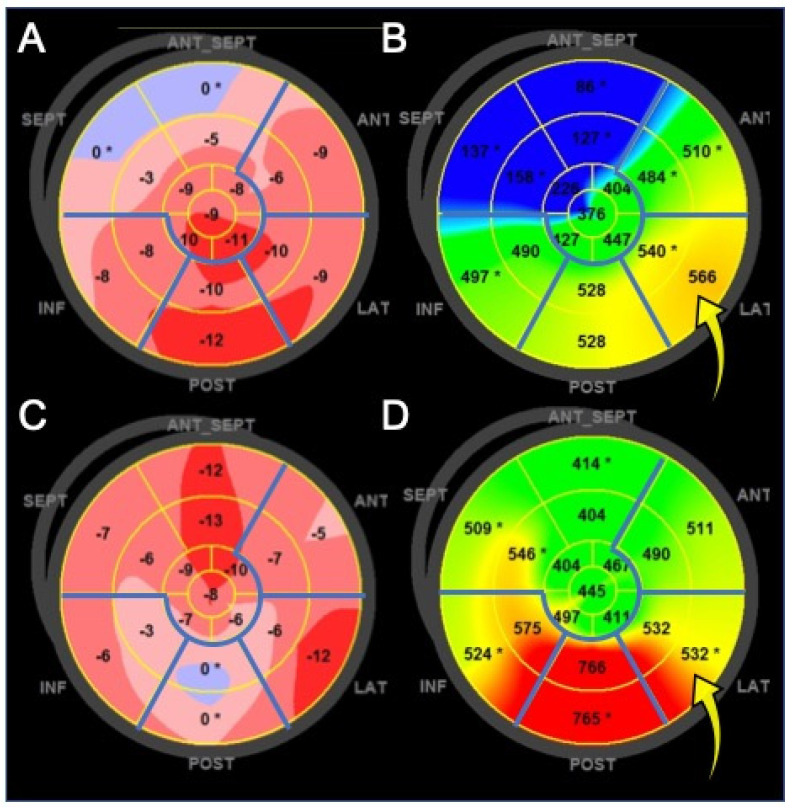
Left ventricle (LV) color-coded polar maps of longitudinal myocardial strain. Panels on the left: map of strain amplitude. Clear pink and blue colors identify the most dysfunctional areas. Panels on the right: map of time-to-peak strain (dyssynchrony map). The red color represents the latest contracting myocardial areas. Basal and mid myocardial segments of inferior, posterior, lateral, and anterior walls (demarcated by the blue lines) are potential sites for LV pacing. (**A**,**B**) Patient with viable myocardium at the basal lateral wall, which is the site with latest contraction delay (optimal pacing site, yellow arrow). (**C**,**D**) The posterior wall is the most dyssynchronous, as indicated by the red color on the dyssynchrony map, but it is not the optimal pacing site because of the very low contraction amplitude. The best site for LV pacing is the lateral wall, although it is not the most delayed (yellow arrow).

One of the promising areas of the new ultrasound technologies in CRT is the optimization of the LV electrode implantation, in order to identify the most appropriate LV site to pace, avoiding stimulation of transmural scar tissue [39]. Patients with LV lead placed in scar-free regions have better survival and less HF hospitalizations than patients with LV lead located in scarred regions [44,53,54]. Two randomized, single center studies, the TARGET and STARTER, proved that patients with LV lead implantation guided by echocardiography (LV lead was placed into a non-scarred LV segment with the most delayed mechanical activation, evaluated by radial STE) had better LV remodeling and better outcome than patients with routine LV lead implantation [55,56]. This was observed also using longitudinal STE [57] (Figure 4).

Table 2 summarizes the most used, STE-derived measures of LV dyssynchrony and discoordination.

The role of intracardiac flow dynamics in predicting CRT response or guiding the site of LV lead implantation is not yet clear [58]. It has been shown, using the Echo-PIV technique, that in CRT responders, when pacing is active, there is a longitudinal alignment (along the main axis of the LV) of the hemodynamic forces associated with intracardiac flow, as it occurs in a normal heart. Pacing switch-off determines the loss of alignment of intraventricular forces, and the development of transversal components with no propulsive function, despite cardiac contractility and synchrony parameters do not show measurable changes [59,60]. Conversely, among CRT non-responders, flow is neither aligned when the pacemaker is active nor when it is switched off. There are also differences in vortex shape and energetic properties between CRT responders and non-responders [61].

More investigations are needed before the utilization of intracardiac flow analysis can be extensively proposed for clinical practice.

## 5. Conclusions

Standard TTE is the most widely used imaging modality for the assessment of patients affected by HF with both reduced and preserved EF, due to its unmatched ability to combine safety and ease of application with depth of diagnostic and prognostic information. LV myocardial deformation study by STE and intracardiac flow analysis by Echo-PIV and CDFM provided valuable insights concerning LV mechanics and hemodynamic forces of patients affected by HF. A promising area for clinical application of these new ultrasound technologies is the selection of CRT responders and the optimization of LV lead delivery. These technological advancements revealed to be encouraging to further expand the role of echocardiography as modality of choice in HF diagnosis, therapeutic decision making, and monitoring of response to treatment.

## Figures and Tables

**Table 1:** Diagnostic and prognostic role of strain pattern according to HF phenotypes.

HF Phenotype	LV Damage	Deformation Imaging	Diagnostic Value of GLS	Prognostic Value of GLS
HFpEF	Subendocardial	Longitudinal dysfunction	<16% (minor criteria) [12]	Predictor of poor prognosis when impaired (HF hospitalization, CV death, cardiac arrest) [8,13,14,15]
HFrEF	Transmural	Longitudinal and circumferential dysfunction	-	Predictor of poor prognosis when impaired (all-cause mortality, ventricular arrhythmias) [16,17,18] Early marker of response to therapy for HF [19]

CV: cardiovascular; GLS: global longitudinal strain; HF: heart failure; HFpEF: heart failure with preserved ejection fraction; HFrEF: heart failure with reduced ejection fraction; LV: left ventricular.

**Table 2:** Some of the new ultrasound technologies-derived measures for evaluation of LV mechanical dyssynchrony and discoordination.

Index	Cut-Off Value	Imaging Method	Echocardiographic View	Parameter to Evaluate
ASPWD	130 ms	2D STE	PSAX (papillary muscles)	LV radial dyssynchrony
SDI	25%	2D STE	Apical 4-, 2-, and 3-C	LV longitudinal dyssynchrony
Tε-SD (MDI)	60 ms	2D STE	Apical 4-, 2-, and 3-C	LV longitudinal dyssynchrony
Septal flash	130 ms	2D STE	Apical 4-C	LV discoordination/ anomalous wall movements
Apical rocking	9.8%	2D STE	Apical 4-C or 3-C	LV discoordination/ anomalous wall movements
RDI	>40%	2D STE	PSAX (papillary muscles)	LV radial discoordination
SRS	4.7%	2D STE	Apical 4-C	LV discoordination/ anomalous wall movements

ASPWD: anteroseptal-posterior wall delay; LV: left ventricular; LVPEP: left ventricular pre-ejection period; MDI: mechanical dispersion index; PLAX: parasternal long-axis; PSAX: parasternal short-axis; PW: pulsed wave; RDI: radial discoordination index; SDI: strain delay index; SPWMD: septal to posterior wall motion delay; SRS: septal rebound stretch; STE: speckle tracking echocardiography; TDI: tissue Doppler imaging; T-SD: time to peak longitudinal strain standard deviation; 2D: two-dimensional; 2-C: 2-chamber; 3-C: 3-chamber; 4-C: 4-chamber; 5-C: 5-chamber.

## Data Availability

Not applicable.
